# DNA Microarray Analysis of Submandibular Glands in IgG4-Related Disease Indicates a Role for MARCO and Other Innate Immune-Related Proteins

**DOI:** 10.1097/MD.0000000000002853

**Published:** 2016-02-18

**Authors:** Miho Ohta, Masafumi Moriyama, Takashi Maehara, Yuka Gion, Sachiko Furukawa, Akihiko Tanaka, Jun-Nosuke Hayashida, Masaki Yamauchi, Noriko Ishiguro, Yurie Mikami, Hiroto Tsuboi, Mana Iizuka-Koga, Shintaro Kawano, Yasuharu Sato, Tamotsu Kiyoshima, Takayuki Sumida, Seiji Nakamura

**Affiliations:** From the Section of Oral and Maxillofacial Oncology, Division of Maxillofacial Diagnostic and Surgical Sciences (MO, MM, TM, SF, AT, J-NH, MY, NI, YM, SK, SN); OBT Research Center, Faculty of Dental Science, Kyushu University, Fukuoka, Japan (MM); Department of Pathology, Okayama University Graduate School of Medicine, Dentistry and Pharmaceutical Sciences, Okayama, Japan (YG, YS); Department of Internal Medicine, Faculty of Medicine, University of Tsukuba, Tsukuba, Japan (HT, MI-K); and Laboratory of Oral Pathology, Division of Maxillofacial Diagnostic and Surgical Sciences, Faculty of Dental Science, Kyushu University (TK), Fukuoka, Japan.

## Abstract

IgG4-related disease (IgG4-RD) is a novel systemic disease entity characterized by elevated serum IgG4 and tissue infiltration of IgG4-positive plasma cells accompanied by severe fibrosis. Although recent studies demonstrated that innate immune cells including monocytes and macrophages might promote local fibrosis and IgG4 production, the pathological mechanism remains unclear. In this study, we sought to identify the disease-associated genes, especially innate immune molecules.

Gene expression was analyzed by DNA microarray in submandibular glands (SMGs) from patients with IgG4-RD (n = 5), chronic sialoadenitis (CS) (n = 3), and controls (n = 3). Differentially expressed genes (DEGs) were validated by real-time polymerase chain reaction (PCR) and immunohistochemical staining in IgG4-RD (n = 18), CS (n = 4), Sjögren syndrome (n = 11), and controls (n = 10).

Gene expression patterns in the 3 groups were quite different from each other by the pvclust method and principal components analysis. In IgG4-RD, 1028 upregulated genes and 692 downregulated genes were identified as DEGs (*P* < 0.05). Gene Ontology (GO) term analysis indicated that the upregulated DEGs in IgG4-RD encoded proteins involved in T/B cell activation and chemotaxis. PCR validated significantly higher expression of macrophage receptor with collagenous structure (MARCO), a pattern-recognition receptor, in IgG4-RD compared with the other groups (*P* < 0.01). Immunohistochemical analysis confirmed that the expression pattern of MARCO was similar to that of the M2 macrophage marker CD163.

MARCO was identified as a disease-associated molecule in IgG4-RD by DNA microarray. Moreover, M2 macrophages might contribute to the initiation of IgG4-RD via MARCO.

## Abstract 2

Supplemental Digital Content is available in the text

## INTRODUCTION

IgG4-related disease (IgG4-RD) is a systemic disease presenting in multiple target organs, including pancreas, salivary and lacrimal glands, lung, thyroid, liver, kidney, aorta, prostate, retroperitoneum, and lymph nodes.^1,2^ This disease is characterized by elevated serum IgG4 concentration and swelling of affected organs with marked infiltration of IgG4- positive plasma cells, hyperplastic ectopic germinal center (GC) formation and severe fibrosis. IgG4-related dacryoadenitis and sialoadenitis (IgG4-DS), so-called Mikuliçz disease, has been considered to be a subtype of Sjögren syndrome (SS) based on the histopathological similarities in glandular tissue. However, in 2005, Yamamoto et al^3^ reported that Mikuliçz disease demonstrated high serum IgG4 concentration and the infiltration of IgG4-positive plasma cells in glandular tissue, and was distinguishable from SS.

Regarding the immunological aspects of this disease, it is well known that IgG4 is induced by T helper type 2 (T_h_2) cytokines such as interleukin (IL)-4 and IL-13.^4^ We previously reported that specific T_h_ cytokines, including IL-4, IL-10, and IL-21, contributed to IgG4 production and ectopic GC formation in IgG4-DS.^5,6^ In addition, recent studies indicated that abnormal innate immune responses enhanced T_h_2 immune responses and the immunopathogenesis of IgG4-RD via toll-like receptors (TLRs) expressed by macrophages.^7^ Our recent data indicated that M2 macrophages might promote T_h_2 inflammatory responses via IL-10 and CC chemokine ligand (CCL) 18 production.^8^ Although macrophages might play an effective role in IgG4 production and are involved in the initiation of IgG4-RD, the pathogenic mechanism of IgG4-RD by innate immune reaction remains unclear.

In this study, we thus sought to identify disease-associated genes, especially innate immune molecules, in IgG4-RD using DNA microarray analysis. As reasonable samples for DNA microarray in this study, we selected submandibular glands (SMGs) because labial salivary glands (LSGs) in IgG4-RD show greater variability in pathology even from the same patient and less characteristic histological findings, such as severe fibrosis and ectopic GC formation, than SMGs.^9^

## MATERIALS AND METHODS

### Patients

For DNA microarray analysis, SMG samples were obtained from 6 patients with IgG4-RD (4 males and 2 females; mean age ± standard deviation (SD), 68.2 ± 5.0 years), 3 patients with chronic sialoadenitis (CS) caused by sialolith (1 male and 2 females; 51.5 ± 21.3 years), and 3 patients with oral squamous cell carcinoma (OSCC) as a control group (3 males; 55.7 ± 11.1 years) who were referred to the Department of Oral and Maxillofacial Surgery, Kyushu University Hospital between 2010 and 2015. The patients underwent the following procedures: open SMG biopsies for IgG4-DS as described by Moriyama et al^9^; submandibulectomy for CS; and neck dissection for OSCC. IgG4-DS was diagnosed according to both the “comprehensive diagnostic criteria for IgG4-related disease” and “diagnostic criteria for IgG4-DS.”^10^ All patients had never been treated with corticosteroids or any other immunosuppressants before SMG biopsy. All SMG samples from patients with IgG4-RD showed characteristic histopathological findings including marked infiltration of IgG4-positive plasma cells, severe fibrosis, and formation of multiple ectopic GCs. In contrast, SMGs from OSCC patients were histologically normal and lacked clinical evidence of metastasis and radiation therapy (see Supplementary Figure 1).

This study design was approved by the Ethics Committee of Kyushu University, Japan, and written informed consent was obtained from all of the patients and healthy controls before inclusion in this study (IRB serial number: 25-287).

### Total RNA Isolation

Total RNA was isolated from SMGs using TRIzol Reagent (Invitrogen Corp., Life Technologies, Carlsbad, CA) and then purified using SV Total RNA Isolation and RNeasy system (Promega Corp., Madison, WI) according to the manufacturer's instructions. RNA samples were quantified using a NanoDrop ND-1000 spectrophotometer (Thermo Fisher Scientific, Inc., Wilmington, DE) and the quality of the RNA was confirmed with an Experion automated electrophoresis station (Bio-Rad Laboratories, Inc., Hercules, CA).

### Gene Expression Microarrays

According to manufacturers’ instructions, the complementary RNA was amplified and labeled by Low Input Quick Amp Labeling Kit (Agilent Technologies, Santa Clara, CA), and hybridized to SurePrint G3 Human Gene Expression Microarrays 8 × 60K v2 (Agilent Technologies) (DNA chip including 60,000 genes). All hybridized microarray slides were scanned using an Agilent scanner. Relative hybridization intensities and background hybridization values were calculated using Agilent Feature Extraction Software (ver. 9.5.1.1).

### Data Analysis and Filter Criteria

The raw signal intensities of all specimens were log_2_-transformed and normalized by the quantile algorithm with “preprocessCore” library package^11^ on Bioconductor software.^12^ We selected the probes that called “Present” flag in at least one sample and used them to extract differentially expressed genes (DEGs), and then applied Linear Models for Microarray Analysis (limma) package of Bioconductor software, and obtained DEGs. To identify up- and downregulated genes, we calculated intensity based *P* values and ratios from the quantile-normalized signal levels of each probe and compared IgG4-RD and CS patients. Then, we established criteria for regulated genes: upregulated genes, ratio ≥4.0-fold and *P* < 0.05; downregulated genes, ratio ≤0.25 and *P* < 0.05. The hierarchical clustering by the pvclust method^13^ and principal components analysis (PCA) were applied to the DEGs from comparison with IgG4-RD and CS. To determine significantly over-represented Gene Ontology (GO) term categories and significant enrichment of pathways (Enrichment Score >1.3), we used GO annotation and the Web tool, the Database for Annotation, Visualization and Integrated Discovery (DAVID) provided by National Institute of Allergy and Infectious Diseases (http://david.abcc.ncifcrf.gov/home.jsp).^14,15^ The TreeMap was generated by REVIGO^16^ to visualize significant (*P* < 0.01) GO terms that were obtained from the Functional Annotation Chart of Biological Process terms (BP_FAT category) on DAVID.

### Validation of the Microarray Results

Quantitative polymerase chain reaction (PCR) and immunohistochemical staining (IHC) were performed to validate the results of DNA microarray analysis. Total RNA was extracted from SMG samples from 18 patients with IgG4-RD, 11 patients with SS, 3 patients with CS, and 10 controls. SS was diagnosed according to both the Research Committee on SS of the Ministry of Health and Welfare of the Japanese Government (1999)^17^ and the American-European Consensus Group criteria for SS.^18^ Each patient showed objective evidence of salivary gland involvement based on the presence of subjective xerostomia and a decreased salivary flow rate, abnormal findings on parotid sialography, and focal lymphocytic infiltrates in the LSGs. There was no documented history of treatment with steroids or any other immunosuppressants, infection with HIV, HTLV-1, hepatitis B virus, or hepatitis C virus, or sarcoidosis in any of the patients. None of the patients had evidence of malignant lymphoma at the time of the study.

### Extraction of RNA and Synthesis of Complementary DNA (cDNA)

Total RNA was isolated from the whole SMG samples by the acidified guanidinium-phenol-chloroform method. Total RNA samples (1 μg) were prepared and used for the synthesis of cDNA and then RNA was incubated for 1 hour at 42°C with 20 U of RNase inhibitor (Promega Corp.), 0.5 μg of oligo-1218 (Pharmacia, Uppsala, Sweden), 0.5 mM of each deoxyribonucleotide triphosphate (dNTP) (Pharmacia), 10 mM of dithiothreitol (DTT), and 100 U of RNA reverse transcriptase (Life Technologies, Gaithersburg, MD).

### Quantitative Estimation of mRNA by Real-Time PCR

The resulting cDNA was amplified using Light Cycler Fast Start DNA Master mix SYBR Green III (Roche Diagnostics, Mannheim, Germany) in a Light Cycler real-time PCR instrument (version 3.5; Roche Diagnostics). The mRNA levels of CCL18, chitinase 1 (CHIT1), macrophage receptor with collagenous structure (MARCO), and TLR8 were analyzed. The primer sequences used were as follows: CCL18 (187 bp), forward 5′-AGCTCTGCTGCCTCGTCTAT-3′, reverse 5′-CAGGCATTCAGCTTCAGGTC-3′; CHIT1 (102 bp), forward 5′-ATATGCCAGTGGGCAGTTTC-3′, reverse 5′-CCTGTTTCCAGGCTTCTGAG-3′; MARCO (122 bp), forward 5′-CAATGTTCCAAAGCCCAAGAG-3′, reverse 5′-CCTGCAGATTCAGAACTTGGA-3′; TLR8 (124 bp), forward 5′-AAGCACATCCCAAATGAAGC-3′, reverse 5′-GCAACTCGAGACGAGGAAAC-3′; β-actin (260 bp), forward 5′-GCAAAGACCTGTACGCCAAC-3′, reverse 5′-CTAGAAGCATTTGCGGTGGA-3′. The relative mRNA levels were calculated after normalizing to the housekeeping gene β-actin.

### Immunohistochemical Analysis

Serial 4-μm-thick sections of SMGs were cut from the block of formalin-fixed and paraffin-embedded tissue and stained with a conventional avidin–biotin complex technique as previously described.^5^ Antibodies used included anti-CD68 (catalog #ab955; Abcam, Cambridge, MA), anti-CD163 (catalog # NCL-CD163; Leica Biosystems, Nussloch GmbH, Germany), and anti-CD123 (catalog # NCL-L-CD123; Leica Biosystems) mouse monoclonal antibodies; anti-CD11c (catalog #52632; Abcam) and anti-CD21 (catalog #ab75985; Abcam) rabbit monoclonal antibodies and a rabbit anti-MARCO polyclonal antibody (catalog #AP9891A; ABGENT, San Diego, CA). Tissue sections were sequentially incubated with primary antibodies for 2.5 hours then with biotinylated anti-mouse IgG or anti-rabbit IgG secondary antibodies (Vector Laboratories, Burlingame, CA), avidin–biotin–horseradish peroxidase complex (Vector Laboratories), and 3,3′-diaminobenzidine (Vector Laboratories). Mayer hematoxylin was used for counterstaining. Photomicrographs were obtained using a light microscope equipped with a digital camera (BZ-9000 series; Keyence, Tokyo, Japan).

### Double Immunohistochemical Analysis

For double immunohistochemical analysis, the formalin-fixed and paraffin-embedded sections of SMGs were immunohistochemically stained using an automated Bond Max stainer (Leica Biosystems). The following primary antibodies were used: anti-CD163 (catalog # NCL-CD163; Leica Biosystems) and anti-MARCO (catalog #AP9891A; ABGENT). Images were taken using a Keyence BZ-9000 series microscope.

### Statistical Analysis

The significance of differences between groups was determined using χ^2^ test, Student *t* test, Mann–Whitney *U* test, Kruskal–Wallis tests, and Spearman rank correlations. A *P*-value of *P* < 0.05 was considered statistically significant. All statistical analyses were performed using JMP software (V.8; SAS Institute, Cary, NC).

## RESULTS

### Identification of Gene Expression Patterns in Patients With IgG4-RD, CS, and Controls

**FIGURE 1:**
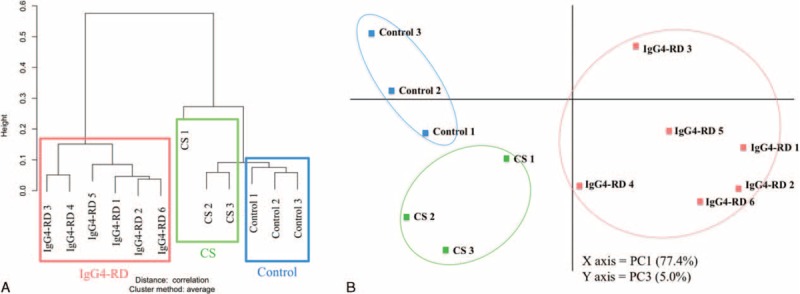
Gene expression patterns in patients with IgG4-related disease, chronic sialoadenitis, and controls. Hierarchical clustering (A) and principal components (PC) analysis (B) by using the quantile-normalized data.

Table 1 summarizes the clinical and pathological features of participating patients and controls analyzed by DNA microarray. Microarray analysis of 60,000 gene expression changes was performed to clarify the gene expression profiles of SMG samples from patients with IgG4-RD, CS, and controls. The scatterplot analysis and heat map to elucidate and visualize the differences in gene expression between IgG4-RD and CS patients showed statistically significantly differences (see Supplementary Figure 2). Figure 1 shows hierarchical clustering by the pvclust method (Figure 1A) and PCA (Figure 1B) using the quantile algorithm-normalized data. The gene expression patterns in the 3 groups were quite different by hierarchical clustering as well as by PCA. Although the 2 patients with CS were located near the control cluster, the IgG4-RD group was different from the other 2 groups by hierarchical clustering. Furthermore, the gene expression patterns of the 3 groups were divided into 3 different clusters by PCA. The contribution of principal component (PC) 1 was 77.4%, and that of PC3 was 5.0%.

**TABLE 1:**
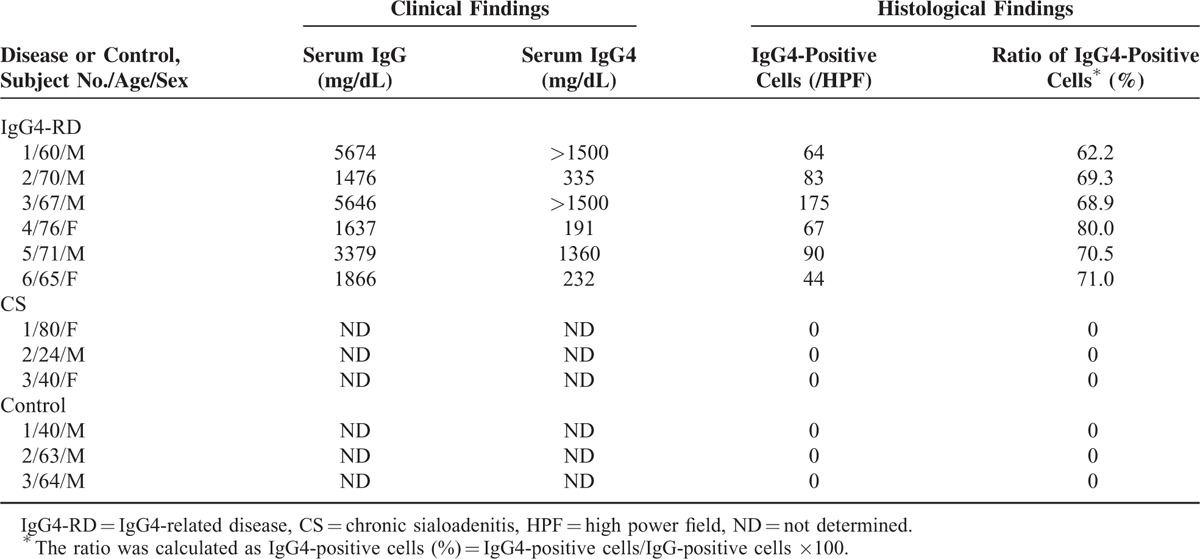
Backgrounds of Patients With IgG4-Related Disease, Chronic Sialoadenitis, and Controls for DNA Microarray Analysis of Submandibular Glands

Once we determined that gene expression patterns in patients with IgG4-RD and CS differed from those in controls by clustering analysis, we next compared gene expression and identified genes differentially expressed between IgG4-RD and CS in pairwise comparisons. A total of 1028 genes were identified as upregulated in IgG4-RD compared with CS by the rank products method (see Supplementary Table 1). In contrast, 692 genes were identified as downregulated in IgG4-RD compared with CS (see Supplementary Table 2).

### Gene Annotation Enrichment Analysis of DEGs by GO Annotation

Significantly enriched GO terms were found in the 1720 genes extracted as up- and downregulated DEGs in IgG4-RD compared with CS as analyzed by the web tools DAVID. Of the 266 different categories by Gene annotation enrichment analysis and the TreeMap, the following 16 categories were identified as involved in IgG4-RD; immune responses, cell cycle, regulation of leukocyte proliferation, nuclear division, macrophage, cell–cell signaling, cell activation, cell adhesion, DNA replication, ectoderm development, organelle localization, microtubule-based process, di- or tri-valent inorganic cation homeostasis, biological adhesion, cellular di- or tri-valent inorganic cation homeostasis, mitosis (see Supplementary Figure 3).

### Validation of Candidate Genes by Quantitative PCR

Several genes with GO terms including immune, inflammatory, or macrophage were in the top 120 upregulated DEGs in IgG4-RD compared with CS (Supplementary Table 1). We chose 4 innate immunity-related genes (CCL18 [rank 3], CHIT1 [rank 25], MARCO [rank 34], and TLR8 [rank 117]) and validated these genes using real-time PCR. β-actin was an appropriate internal control because the mRNA expression level of β-actin was not significantly different between IgG4-RD and CS in the DNA microarray analysis.

**FIGURE 2:**
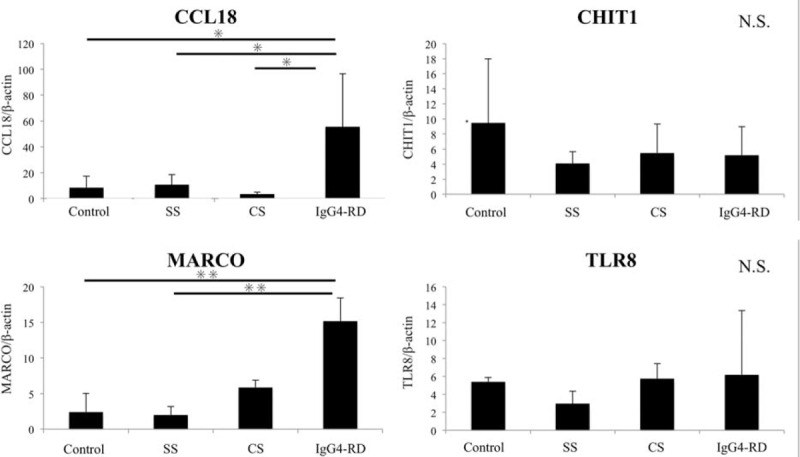
Quantitative PCR analysis for validation. Quantitative PCR analysis was performed using salivary glands from the patients with IgG4-RD and chronic sialoadenitis (CS), Sjögren syndrome (SS), and controls. The bar shows the mean value ± standard deviation. Significant differences between groups were determined by the Kruskal–Wallis test (^∗^*P* < 0.05, ^∗∗^*P* < 0.01). NS, not significant.

As shown in Figure 2, the mRNA expression levels of CHIT1 and TLR8 showed no statistically significant difference between groups. In contrast, the mRNA expression levels of CCL18 and MARCO in the SGs from patients with IgG4-RD were significantly higher than those from the other groups. Our previous reports^8^ already demonstrated that CCL18 secreted preferentially by M2 macrophages promotes the development of severe fibrosis in IgG4-DS. In this study, we therefore focused on MARCO as a disease-related molecule.

### Expression of MARCO in SMGs

**FIGURE 3:**
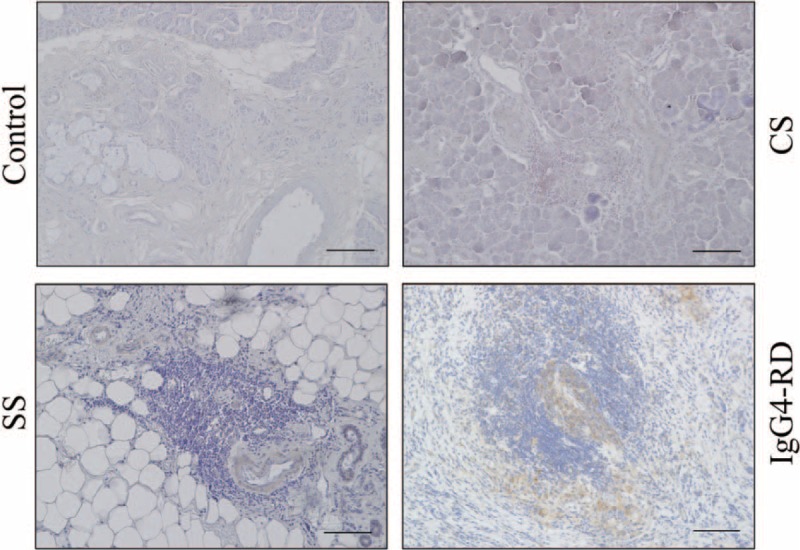
Expression of MARCO in submandibular glands. Immunohistochemical staining with MARCO in submandibular glands (SMGs) from representative patients with IgG4-RD, CS, SS, and controls. Counterstaining was performed with Mayer hematoxylin (blue). Scale bars, 100 μm.

The SMG samples from patients with IgG4-RD, CS, and SS, and controls were immunohistochemically examined to evaluate the distribution of MARCO. Expression of MARCO could be strongly detected around ectopic GCs only in patients with IgG4-RD, but was rarely seen in the other groups (Figure 3).

### Identification of MARCO-Expressing Cells in SMGs From Patients With IgG4-RD

As MARCO is considered to be expressed on both macrophages and dendritic cells (DCs), we next examined the localization of MARCO and the following markers of macrophages and DCs: CD68 as a marker of both M1 and M2 macrophages; CD163 as a marker of M2 macrophages; CD11c as a marker of myeloid DCs; CD123 as a marker of plasmacytoid DCs; and CD21 as a marker of follicular DCs, in SMG from patients with IgG4-RD.

**FIGURE 4:**
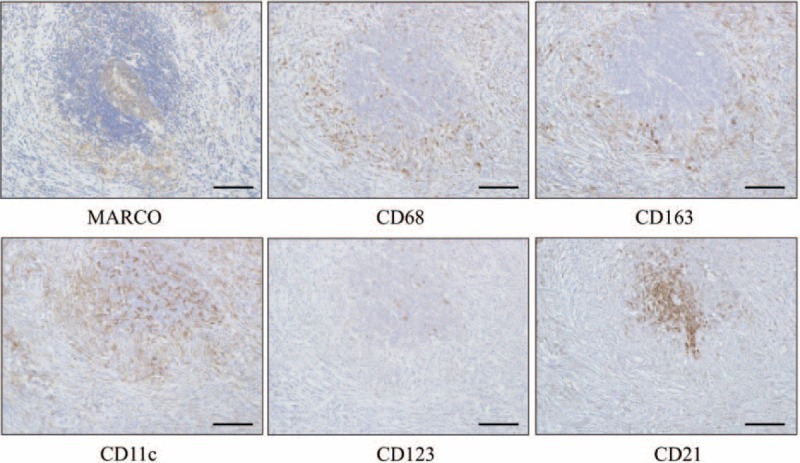
Distribution of MARCO-expressing cells in SMGs from representative patients with IgG4-RD. Each set of serial SMG sections was stained with MARCO, CD68 as a marker of both M1 and M2 macrophages, CD163 as a marker of M2 macrophages, CD11c as a marker of myeloid dendritic cells (DCs), CD123 as a marker of plasmacytoid DCs, and CD21 as a marker of follicular DCs. Counterstaining was performed with Mayer hematoxylin (blue). Scale bars, 100 μm.

As shown in Figure 4, expression of CD11c and CD21 could be detected mainly in ectopic GCs in SG from patients with IgG4-RD, while CD123 reactivity was diffuse and only slightly detected. Interestingly, expression of CD68 and CD163 could be detected around ectopic GCs. The distribution of both of these markers was very similar to that of MARCO.

**FIGURE 5:**
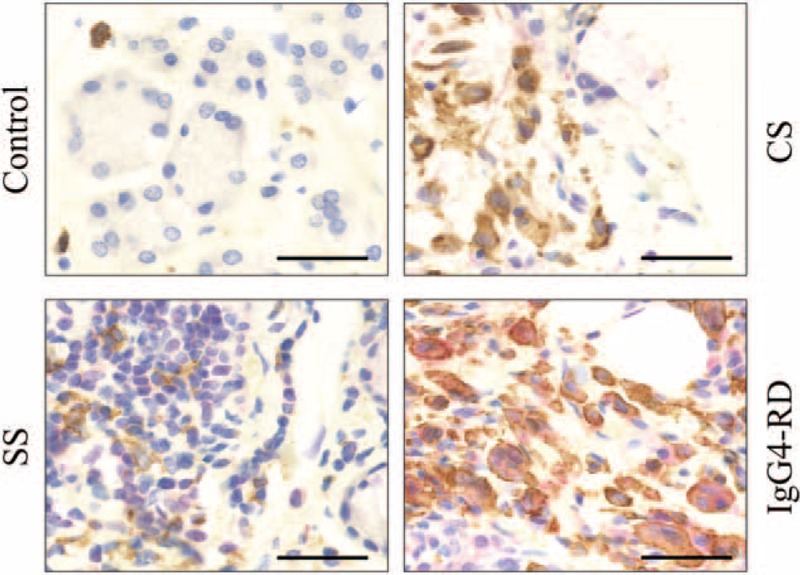
Identification of MARCO-expressing cells in SMGs. Double immunostaining with CD163 (brown) and MARCO (red) in SMGs from representative patients with IgG4-RD, CS, SS, and controls. Counterstaining was performed with Mayer hematoxylin (blue). Scale bar, 50 μm.

As mentioned above, IgG4-RD shows predominant infiltration of M2 macrophages over M1 macrophages in the local lesions. Therefore, to clarify whether M2 macrophages expressed MARCO, double staining with CD163 and MARCO was performed. Notably, CD163-positive cells (brown) were colocalized with MARCO-positive cells (red) only in SMGs from patients with IgG4-RD (Figure 5).

## DISCUSSION

IgG4-RD is a new emerging disease entity that has received much attention recently. Several studies have already reported the clinical features and symptoms of this disease.^1,19,20^ Although IgG4-RD has been considered to be predominantly a T_h_2 immune response-mediated disease^5,21–23^ recent studies indicated that the initiation of IgG4-RD might be caused by not only acquired immunity, but also innate immunity.^24,25^ Watanabe et al^7^ demonstrated that abnormal innate immune responses induced via TLR signaling in monocytes/macrophages might enhance the immunopathogenesis of IgG4-RD. However, the detailed mechanism of pathogenesis by the innate immune system remains to be completely elucidated. Therefore, we performed gene expression microarray analysis to identify innate immune molecules, especially related to macrophages, involved in the initiation of IgG4-RD. We have previously analyzed the gene expression patterns in LSG samples from patients with IgG4-RD and SS by DNA microarray.^26^ However, SS is a unique inflammatory disease similar to IgG4-RD, while CS is a nonspecific inflammatory disease, suggesting that CS is suitable as positive controls for IgG4-RD.

Microarray of SMGs demonstrated significant differences in gene expression patterns among the patients with IgG4-RD, CS, and controls. According to the results of Functional Annotation Clustering on DAVID, we found that the terms related with immune response, inflammatory response, and macrophage were enriched in IgG4-RD compared with CS. Thus, IgG4-RD might be caused by some specific inflammatory milieu and these results are consistent with past reports with regard to the pathogenesis of IgG4-RD.

In this study, overexpression of CCL18 and MARCO in SMGs from patients with IgG4-RD compared with the patients with CS, SS, and controls was validated by real-time PCR. CCL18 was identified previously as a chemokine with fibrotic activity and selective chemotactic activity on peripheral blood T lymphocytes, especially T_h_2 cells.^27^ We have already reported that CCL18 was associated with the development of severe fibrosis in IgG4-RD.^8^ In addition, previous data, indicating that tissue-infiltrating and circulating IgG4-positive cells in IgG4-RD are polyclonal,^28^ suggest that there are many antigen responses in local lesions. Thus, we focused on pattern recognition receptor MARCO as a new candidate molecule related to innate immunity in this study.

MARCO was identified as a pattern-recognition receptor belonging to the scavenger receptors expressed by macrophages,^29^ and is considered to play an important role in the innate immune response by mediating ligand binding and phagocytosis. This receptor can recognize various ligands including bacterial lipopolysaccharide (LPS), acetylated low density lipoprotein (LDL), apoptotic cells, lung pathogens, *Streptococcus* species, and ozone-generated oxidized lipids, environmental particles, and nanomaterials such as carbon nanotube and silica.^30^ The expression of MARCO on alveolar macrophages was reported to be strongly increased by exposure to carbon nanotubes, which were similar in structure to asbestos.^31^ Macrophages are well known as key regulators of the immune system due to recognizing pathogen antigens, phagocytosing them and presenting them to lymphocytes. There are at least 2 major subsets of macrophages; classically activated (M1) macrophages stimulated by T_h_1-type responses and alternatively activated (M2) macrophages stimulated by T_h_2-type responses.^32^.M2 macrophages scavenge debris and contribute to angiogenesis, suppression of adaptive immunity, wound healing and fibrosis by producing IL-10 and CCL18.^33^ Murthy et al^34^ found that alveolar macrophages from patients with asbestos-induced pulmonary fibrosis showed high expression levels of MARCO. These observations suggested that MARCO promotes the polarization to a profibrotic M2 phenotype and causes an excessive fibrotic response to lung injury.

Immunohistochemical analysis in our present study found that MARCO was strongly expressed around ectopic GC only in IgG4-RD patients and was colocalized with CD163-positive cells (M2 macrophages). Our previous data indicated that IgG4-RD patients showed a predominant infiltration of M2 macrophages in multiple lesions including those of the pancreas, pleura, prostate glands, lacrimal glands, and SGs.^8^ In addition, IgG4-RD patients showed strong infiltration of DCs. Several studies reported that increased DCs might play a pathological role in IgG4-RD.^35,36^ However, additional research is required to further elucidate the involvement of these cells in the pathogenesis of IgG4-RD.

Considering the current results, M2 macrophages may contribute to the initiation or maintenance of IgG4-RD via MARCO. We thus made the following hypothesis regarding the pathogenic process in IgG4-RD; M2 macrophages recognize certain exogenous or endogenous molecules through binding to MARCO which promote the production of factors, including IL-10 and CCL18, which precipitate an exaggerated fibrosis and the pathology noted in IgG4-RD (see Supplementary Figure 4).

In conclusion, we have confirmed that MARCO is overexpressed in M2 macrophages clustered around ectopic GCs. However, we need to validate the mRNA expression levels of MARCO and the other DEGs related to innate immunity in many more patients, and perform functional assays to elucidate the relationship between innate immunity and IgG4-RD. A more thorough understanding of the role of innate immune cells in IgG4-RD, especially M2 macrophages, could lead to the establishment of a mouse model of IgG4-RD and the development of novel pharmacological strategies aimed at disrupting the innate immune network and inhibiting the initiation or progression of IgG4-RD.

## Supplementary Material

Supplemental Digital Content
