# Supplementary material for: DNA Microarray Analysis of Submandibular Glands in IgG4-Related Disease Indicates a Role for MARCO and Other Innate Immune-Related Proteins
Source: Medicine (Baltimore). 2016 Feb 18;95(7):e2853. doi: 10.1097/MD.0000000000002853 (PMC4998650; doi:10.1097/MD.0000000000002853)
Supplement: Supplemental Digital Content [file medi-95-e2853-s001.doc]

Supplementary Figure 1. Histological findings in submandibular glands. Staining with haematoxylin and eosin (HE) and IgG4 in SMGs from representative patients with IgG4-related disease, CS, SS, and controls. Counterstainig was performed with Mayer’s hematoxylin (blue). Scale bars, 200░μm.


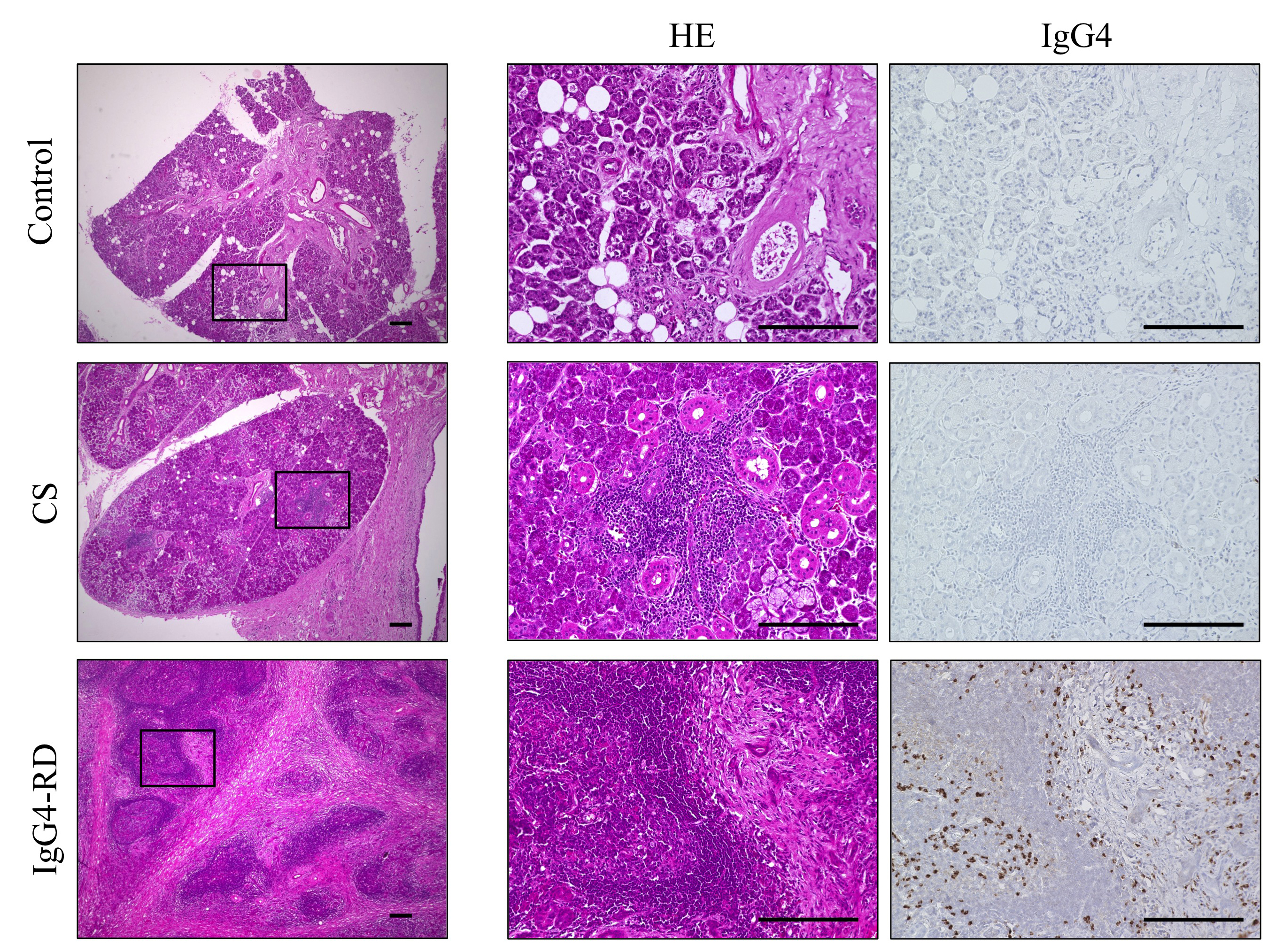


Supplementary Figure 2. Gene expression patterns in patients with IgG4-RD, CS, and controls. (A) Scatterplot analysis to elucidate and visualize the differences in gene expression in patients with IgG4-RD and CS. Pink dots showed up-regulated genes and blue dots showed down-regulated genes in IgG4-RD compared with CS. (B) Heat map showed statistically significantly different in gene expression levels among IgG4-RD, CS, and controls. Colors represented the distance from the median values. Red blocks indicated high, green blocks low, and black blocks the median values.


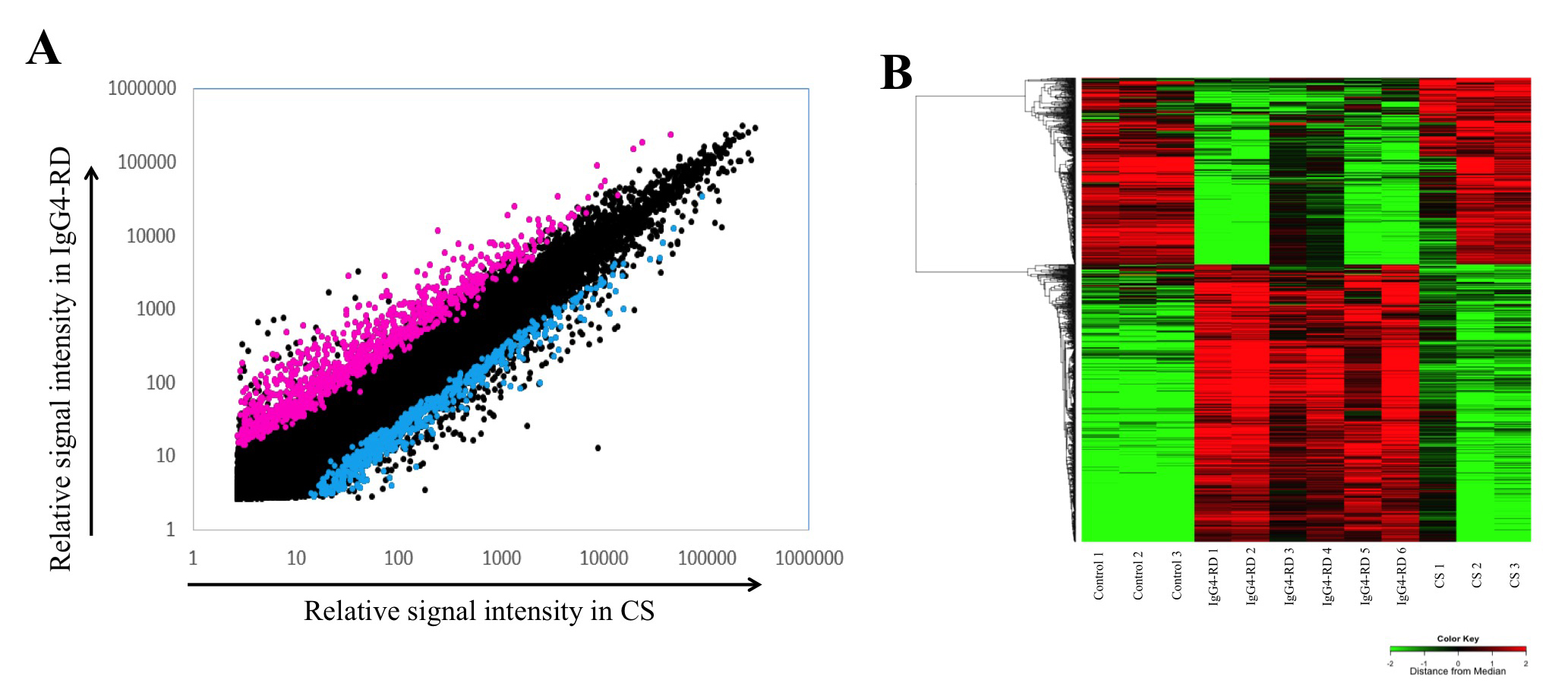


Supplementary Figure 3. Gene annotation enrichiment analysis. The TreeMap was generated by REVIGO program with enriched Gene Ontology (GO) terms that summarized and visualized the biological process of differentially expressed genes in IgG4-RD compared with CS. Eighty-four rectangles reflected the enriched GO term as its size and were divided into the 16 categories by different colors.


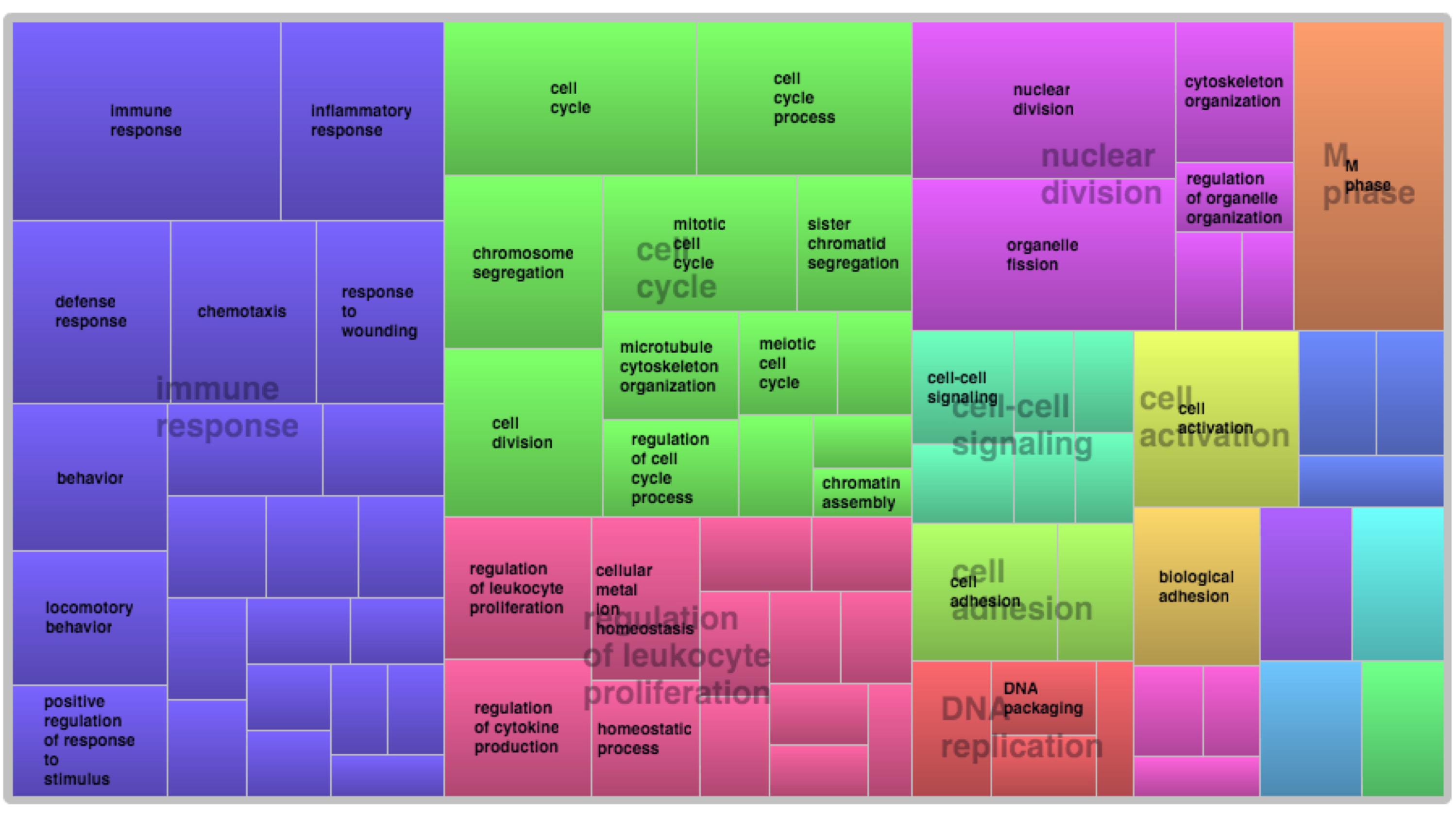


Supplementary Figure 4. Schematic model of innate immune network in IgG4-RD. M2 macrophages recognize some exogenous or endogenous antigens via MARCO and play key roles in local fibrosis and IgG4 production. T helper type 2 cells and M2 macrophages perform positive feedback by producing CCL18 and IL-4.


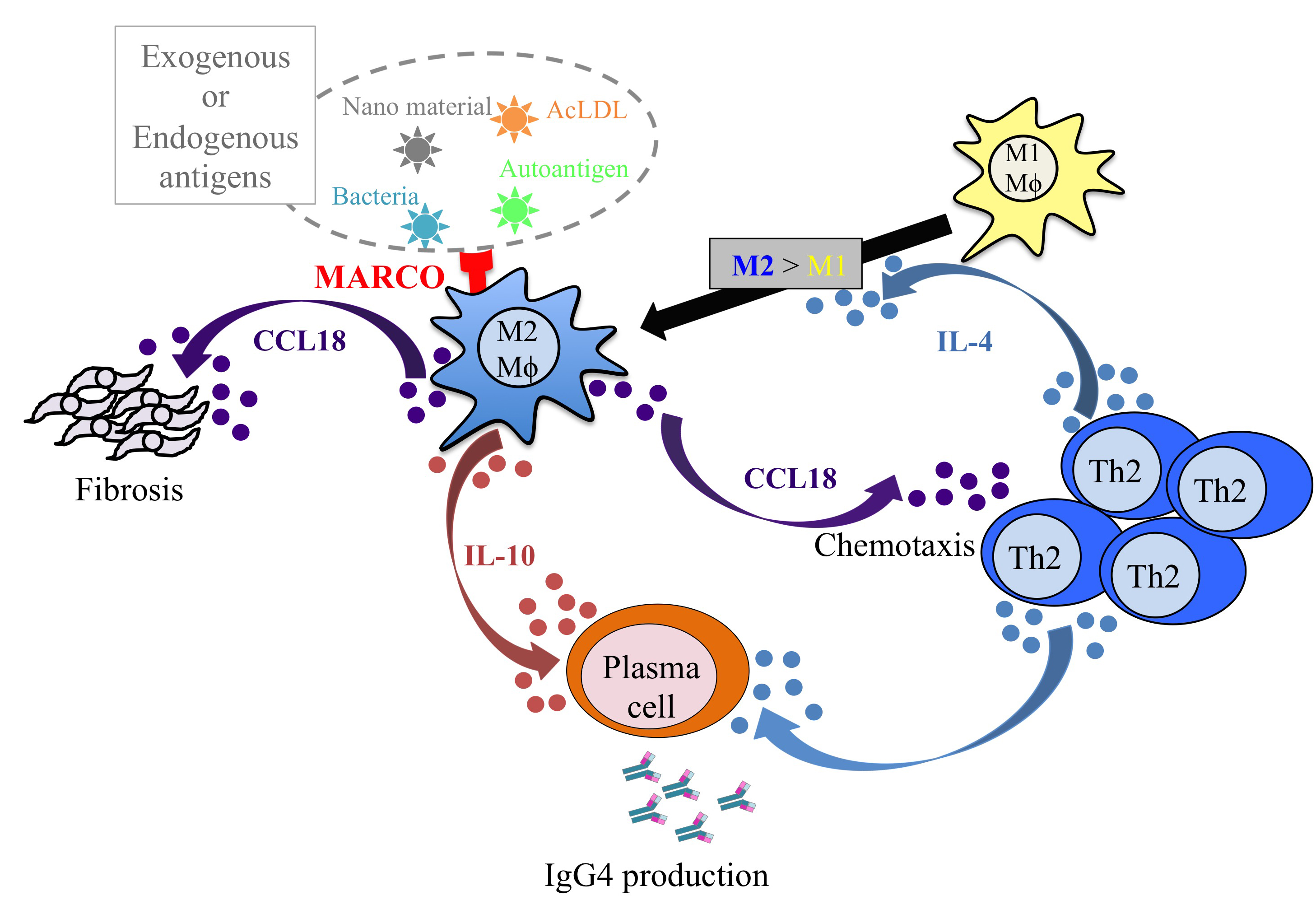


| Rank | Gene Symbol | *p*-value | Ratio | Rank | Gene Symbol | *p*-value | Ratio | | Rank | Gene Symbol | *p*-value | Ratio | |
| --- | --- | --- | --- | --- | --- | --- | --- | --- | --- | --- | --- | --- | --- |
| 1 | AICDA | 9.6×10^-6^ | 47.99 | 41 | CCNA2 | 4.6×10^-5^ | 17.72 | | 81 | CASC5 | 4.6×10^-6^ | 13.01 | |
| 2 | CXCL13 | 1.6×10^-4^ | 45.19 | 42 | SPC25 | 8.2×10^-5^ | | 17.41 | 82 | CCL19 | 1.5×10^-4^ | 13.00 | |
| 3 | CCL18 | 1.5×10^-6^ | 44.01 | 43 | E2F8 | 5.6×10^-6^ | | 17.38 | 83 | TOP2A | 4.6×10^-4^ | | 12.96 |
| 4 | IL21 | 9.7×10^-8^ | 43.09 | 44 | IL4 | 1.2×10^-6^ | | 17.33 | 84 | PDCD1 | 1.5×10^-6^ | 12.93 | |
| 5 | LOC100508196 | 5.3×10^-5^ | 41.98 | 45 | CCNB2 | 5.6×10^-6^ | | 17.16 | 85 | GNG4 | 2.9×10^-4^ | 12.88 | |
| 6 | ZBED2 | 9.0×10^-6^ | 41.05 | 46 | LRMP | 1.0×10^-4^ | | 17.01 | 86 | TPX2 | 3.0×10^-4^ | 12.81 | |
| 7 | NMU | 3.4×10^-7^ | 39.82 | 47 | FAM72D | 3.2×10^-5^ | | 16.82 | 87 | LOC93444 | 2.6×10^-6^ | 12.81 | |
| 8 | TIMD4 | 9.9×10^-6^ | 34.00 | 48 | LOC100130458 | 7.7×10^-5^ | | 16.57 | 88 | APOC1 | 2.1×10^-4^ | 12.72 | |
| 9 | CD5L  HTRA4 | 1.0×10^-3^  6.1×10^-5^ | 32.62  32.23 | 49 | HJURP | 3.0×10^-6^ | | 16.46 | 89 | HIST2H3A | 6.8×10^-4^ | 12.57 | |
| 10 |  |  |  | 50 | CENPM | 1.9×10^-4^ | | 16.39 | 90 | NPL | 7.6×10^-5^ | 12.53 | |
| 11 | TCL1A | 1.3×10^-3^ | 30.74 | 51 | GCET2 | 5.5×10^-4^ | | 16.38 | 91 | STAG3 | 1.9×10^-3^ | 12.39 | |
| 12 | HIST1H2AI | 1.2×10^-4^ | 30.62 | 52 | PMCH | 1.8×10^-4^ | | 16.28 | 92 | MYBL2 | 9.5×10^-5^ | 12.34 | |
| 13 | LOC100507254 | 1.6×10^-5^ | 29.71 | 53 | MKI67 | 4.0×10^-6^ | | 16.18 | 93 | SYNDIG1 | 2.6×10^-3^ | 12.33 | |
| 14 | ALPK2 | 7.8×10^-5^ | 28.48 | 54 | CDKN3 | 8.6×10^-5^ | | 15.75 | 94 | XLOC_006756 | 3.8×10^-4^ | 12.32 | |
| 15 | CR2 | 3.3×10^-4^ | 28.04 | 55 | BUB1B | 3.0×10^-5^ | | 15.60 | 95 | VNN2 | 3.8×10^-4^ | 12.26 | |
| 16 | GTSE1 | 2.3×10^-5^ | 26.80 | 56 | DLGAP5 | 3.1×10^-5^ | | 15.49 | 96 | HMMR | 1.2×10^-4^ | 12.24 | |
| 17 | IL2RA | 3.7×10^-5^ | 26.36 | 57 | ARHGAP11B | 1.3×10^-6^ | | 14.95 | 97 | LAG3 | 1.6×10^-5^ | 12.16 | |
| 18 | RGS13 | 7.2×10^-4^ | 26.16 | 58 | AURKB | 5.5×10^-4^ | | 14.85 | 98 | SIRPG | 6.1×10^-5^ | 12.13 | |
| 19 | HIST1H1B  NUF2 | 1.3×10^-6^  9.4×10^-6^ | 26.15  25.82 | 59 | CD72 | 3.9×10^-5^ | | 14.69 | 99 | MELK | 2.8×10^-4^ | 12.07 | |
| 20 |  |  |  | 60 | NPHS1 | 6.4×10^-4^ | | 14.67 | 100 | TNFRSF9 | 1.5×10^-4^ | 12.00 | |
| 21 | CENPA | 1.0×10^-5^ | 25.74 | 61 | HOXB6 | 1.9×10^-4^ | | 14.65 | 101 | SPP1 | 1.4×10^-2^ | 11.94 | |
| 22 | MMP12 | 5.0×10^-4^ | 25.62 | 62 | CDCA2 | 2.2×10^-5^ | | 14.63 | 102 | SLAMF6 | 7.7×10^-4^ | 11.90 | |
| 23 | BUB1 | 2.4×10^-6^ | 22.52 | 63 | KIF14 | 4.3×10^-6^ | | 14.59 | 103 | IGFL2 | 7.2×10^-4^ | 11.88 | |
| 24 | TM7SF4 | 5.7×10^-3^ | 22.05 | 64 | PBK | 1.2×10^-4^ | | 14.57 | 104 | BIRC5 | 1.0×10^-5^ | 11.87 | |
| 25 | CHIT1 | 6.2×10^-3^ | 21.93 | 65 | CXCR2P1 | 1.0×10^-6^ | | 14.57 | 105 | CTLA4 | 2.7×10^-4^ | 11.79 | |
| 26 | CETP | 3.6×10^-6^ | 21.73 | 66 | LOC340515 | 1.4×10^-3^ | | 14.51 | 106 | KIF4A | 2.4×10^-4^ | 11.76 | |
| 27 | C17orf99  ADAMDEC1 | 8.1×10^-5^  3.7×10^-5^ | 21.57  20.82 | 67 | KIFC1 | 1.4×10^-3^ | | 14.46 | 107 | FAM72A | 2.8×10^-4^ | 11.74 | |
| 28 |  |  |  | 68 | RRM2 | 4.3×10^-6^ | | 14.41 | 108 | LRRC15 | 2.1×10^-2^ | 11.61 | |
| 29 | MMP9 | 2.9×10^-4^ | 20.69 | 69 | SLC2A5 | 4.6×10^-4^ | | 14.41 | 109 | UBE2C | 2.7×10^-5^ | 11.60 | |
| 30 | NLRP4 | 2.4×10^-5^ | 20.67 | 70 | KIF2C | 7.8×10^-5^ | | 14.33 | 110 | POLQ | 3.9×10^-5^ | 11.60 | |
| 31 | DHRS9 | 8.8×10^-6^ | 20.08 | 71 | SPIB | 3.5×10^-3^ | | 14.33 | 111 | BFSP2 | 1.6×10^-4^ | 11.55 | |
| 32 | XLOC_12_015342 | 6.9×10^-6^ | 20.04 | 72 | IGF2BP3 | 3.2×10^-7^ | | 14.19 | 112 | FCRLA | 5.0×10^-3^ | 11.49 | |
| 33 | ASPM | 8.7×10^-6^ | 19.47 | 73 | CXCR5 | 3.5×10^-3^ | | 14.18 | 113 | ESCO2 | 6.1×10^-5^ | 11.47 | |
| 34 | MARCO | 3.6×10^-4^ | 19.38 | 74 | FCRL3 | 1.5×10^-4^ | | 14.02 | 114 | CD22 | 7.0×10^-3^ | 11.45 | |
| 35 | E2F2 | 5.7×10^-6^ | 19.30 | 75 | CENPE | 3.5×10^-5^ | | 13.84 | 115 | KIF15 | 3.3×10^-4^ | 11.39 | |
| 36 | SHCBP1 | 5.8×10^-5^ | 18.84 | 76 | LOC100507055 | 2.4×10^-5^ | | 13.73 | 116 | IL4I1 | 2.9×10^-4^ | 11.27 | |
| 37 | SERPINA9  XLOC_12_013730  GPR18  IL21R | 1.1×10^-3^  1.5×10^-3^  1.3×10^-4^  2.2×10-^4^ | 18.15  18.64  18.54  18.15 | 77 | TIFAB | 1.2×10^-4^ | | 13.65 | 117 | TLR8 | 5.3×10^-5^ | 11.27 | |
| 38 |  |  |  | 78 | CEP55 | 2.9×10^-7^ | | 13.58 | 118 | TNFRSF8 | 6.6×10^-5^ | 11.26 | |
| 39 |  |  |  | 79 | KIF20A | 3.3×10^-4^ | | 13.36 | 119 | FANCA | 3.4×10^-5^ | 11.17 | |
| 40 |  |  |  | 80 | MMP10 | 2.7×10^-2^ | | 13.09 | 120 | CD19 | 8.5×10^-4^ | 11.11 | |

**Supplementary Table 1.** List of top 120 up-regulated differentially expressed genes in IgG4-related disease compared to chronic sialoadenitis.

**Supplementary Table 2.** List of top 120 down-regulated differentially expressed genes in IgG4-related disease compared to chronic sialoadenitis.

| Rank | Gene Symbol | *p*-value | Ratio | Rank | Gene Symbol | *p*-value | Ratio | | Rank | Gene Symbol | *p*-value | Ratio | |
| --- | --- | --- | --- | --- | --- | --- | --- | --- | --- | --- | --- | --- | --- |
| 1 | C6orf58 | 1.9×10^-2^ | 0.010 | 41 | ACTG2 | 1.9×10^-3^ | 0.093 | | 81 | MUC5B | 4.0×10^-4^ | 0.120 | |
| 2 | CSF3 | 2.0×10^-7^ | 0.024 | 42 | RND1 | 3.5×10^-3^ | | 0.093 | 82 | PLIN5 | 4.8×10^-2^ | 0.123 | |
| 3 | KRT6B | 1.6×10^-5^ | 0.032 | 43 | KRT16P2 | 6.2×10^-4^ | | 0.094 | 83 | SLC5A1 | 2.3×10^-3^ | | 0.124 |
| 4 | MYOC | 8.8×10^-3^ | 0.035 | 44 | OVOL1 | 5.8×10^-3^ | | 0.094 | 84 | C10orf81 | 3.7×10^-3^ | 0.124 | |
| 5 | IL8 | 1.8×10^-4^ | 0.043 | 45 | CXCL1 | 3.3×10^-5^ | | 0.094 | 85 | BMPER | 6.5×10^-4^ | 0.124 | |
| 6 | TFF3 | 2.2×10^-2^ | 0.044 | 46 | SERPINB5 | 8.1×10^-3^ | | 0.095 | 86 | PTGER3 | 1.2×10^-2^ | 0.125 | |
| 7 | DLK1 | 4.9×10^-2^ | 0.049 | 47 | KRT42P | 2.2×10^-4^ | | 0.095 | 87 | LOC100128098 | 1.7×10^-2^ | 0.125 | |
| 8 | CXCL2 | 6.4×10^-6^ | 0.054 | 48 | SPINK5 | 7.1×10^-3^ | | 0.097 | 88 | PDE11A | 4.5×10^-3^ | 0.125 | |
| 9 | LECT1  FGFBP1 | 6.8×10^-5^  1.2×10^-4^ | 0.056  0.057 | 49 | AGR2 | 2.5×10^-2^ | | 0.098 | 89 | CGREF1 | 4.6×10^-2^ | 0.125 | |
| 10 |  |  |  | 50 | CNN1 | 1.4×10^-3^ | | 0.098 | 90 | CXCL3 | 3.9×10^-3^ | 0.126 | |
| 11 | KLK8 | 1.1×10^-4^ | 0.059 | 51 | IL17B | 1.4×10^-3^ | | 0.098 | 91 | LOC400128 | 4.8×10^-2^ | 0.126 | |
| 12 | STAC2 | 4.4×10^-4^ | 0.059 | 52 | XLOC_014229 | 3.4×10^-5^ | | 0.099 | 92 | ENTPD8 | 7.0×10^-3^ | 0.127 | |
| 13 | NR4A3 | 1.4×10^-3^ | 0.061 | 53 | KRT17 | 1.2×10^-3^ | | 0.099 | 93 | WNT3A | 4.0×10^-2^ | 0.127 | |
| 14 | KLK7 | 1.7×10^-4^ | 0.062 | 54 | LRRTM1 | 2.6×10^-2^ | | 0.099 | 94 | LDLR | 7.6×10^-7^ | 0.127 | |
| 15 | AGAP1-IT1 | 4.4×10^-6^ | 0.067 | 55 | OXGR1 | 8.0×10^-3^ | | 0.100 | 95 | ABCC6 | 4.1×10^-2^ | 0.128 | |
| 16 | KRT6C | 1.3×10^-9^ | 0.067 | 56 | SPINK6 | 1.9×10^-5^ | | 0.101 | 96 | CCL20 | 3.8×10^-4^ | 0.128 | |
| 17 | SOSTDC1 | 3.6×10^-3^ | 0.068 | 57 | MAFF | 1.1×10^-4^ | | 0.101 | 97 | BPIFA1 | 1.4×10^-2^ | 0.128 | |
| 18 | ATP2C2 | 9.8×10^-3^ | 0.069 | 58 | GLYATL2 | 3.1×10^-4^ | | 0.102 | 98 | LOC286058 | 2.7×10^-4^ | 0.129 | |
| 19 | WIF1  GRIN2C | 1.1×10^-2^  2.3×10^-2^ | 0.071  0.071 | 59 | CA4 | 2.6×10^-3^ | | 0.105 | 99 | NRK | 9.7×10^-5^ | 0.129 | |
| 20 |  |  |  | 60 | SOX11 | 6.2×10^-7^ | | 0.107 | 100 | CLDN10 | 3.1×10^-3^ | 0.129 | |
| 21 | DNER | 3.8×10^-2^ | 0.073 | 61 | C10orf90 | 3.7×10^-2^ | | 0.108 | 101 | SNORD114-20 | 5.6×10^-4^ | 0.130 | |
| 22 | XLOC_012564 | 2.2×10^-2^ | 0.073 | 62 | NPY1R | 1.2×10^-3^ | | 0.108 | 102 | C4orf19 | 3.3×10^-2^ | 0.130 | |
| 23 | LOC100506767 | 4.1×10^-2^ | 0.075 | 63 | PRRT1 | 2.7×10^-2^ | | 0.108 | 103 | CLDN4 | 8.3×10^-3^ | 0.130 | |
| 24 | SCRG1 | 4.5×10^-4^ | 0.076 | 64 | SCN2B | 2.1×10^-3^ | | 0.110 | 104 | DFNB31 | 3.5×10^-3^ | 0.132 | |
| 25 | CCDC129 | 1.1×10^-2^ | 0.076 | 65 | AREG | 9.1×10^-4^ | | 0.110 | 105 | XLOC_002867 | 2.0×10^-3^ | 0.132 | |
| 26 | ATF3 | 2.3×10^-3^ | 0.076 | 66 | PTK7 | 3.5×10^-2^ | | 0.110 | 106 | RPL13AP17 | 4.4×10^-2^ | 0.132 | |
| 27 | KANK4  DMBT1 | 1.9×10^-2^  4.8×10^-2^ | 0.077  0.078 | 67 | SLC6A14 | 8.0×10^-3^ | | 0.113 | 107 | KCNS1 | 2.4×10^-3^ | 0.133 | |
| 28 |  |  |  | 68 | C2orf40 | 1.1×10^-3^ | | 0.113 | 108 | TCOF1 | 4.7×10^-2^ | 0.133 | |
| 29 | OR4D10 | 4.2×10^-2^ | 0.078 | 69 | IL6 | 1.7×10^-3^ | | 0.114 | 109 | SFTPA2 | 5.4×10^-3^ | 0.133 | |
| 30 | KLK5 | 1.5×10^-4^ | 0.080 | 70 | BPIFB2 | 3.6×10^-2^ | | 0.114 | 110 | LOC149773 | 4.9×10^-2^ | 0.133 | |
| 31 | XDH | 2.9×10^-5^ | 0.080 | 71 | CA3 | 8.7×10^-3^ | | 0.114 | 111 | KAZALD1 | 5.0×10^-4^ | 0.133 | |
| 32 | LOC100505851 | 4.4×10^-5^ | 0.080 | 72 | LCN2 | 1.2×10^-2^ | | 0.115 | 112 | TAC1 | 2.6×10^-4^ | 0.134 | |
| 33 | FMO6P | 1.7×10^-2^ | 0.083 | 73 | LINC00473 | 8.3×10^-3^ | | 0.115 | 113 | LRRD1 | 2.3×10^-2^ | 0.134 | |
| 34 | VTCN1 | 1.8×10^-4^ | 0.085 | 74 | LRP1B | 4.9×10^-3^ | | 0.116 | 114 | CACNB2 | 4.8×10^-2^ | 0.134 | |
| 35 | ROPN1 | 4.3×10^-2^ | 0.087 | 75 | IRX1 | 1.4×10^-3^ | | 0.116 | 115 | TFAP2B | 1.3×10^-2^ | 0.134 | |
| 36 | XLOC_l2_013052 | 5.4×10^-5^ | 0.089 | 76 | ARG2 | 3.6×10^-5^ | | 0.117 | 116 | PLXNB3 | 2.4×10^-2^ | 0.134 | |
| 37 | AMTN  BPIFB1  PRSS21  KRT14 | 1.1×10^-3^  1.9×10^-3^  1.5×10^-2^  1.5×10^-3^ | 0.090  0.091  0.092  0.092 | 77 | SCGB1D1 | 3.6×10^-2^ | | 0.118 | 117 | SLC34A2 | 2.4×10^-3^ | 0.135 | |
| 38 |  |  |  | 78 | XLOC_l2_006021 | 1.6×10^-4^ | | 0.119 | 118 | CNTNAP3B | 2.5×10^-3^ | 0.136 | |
| 39 |  |  |  | 79 | FOSB | 2.0×10^-2^ | | 0.119 | 119 | ADAMTS4 | 1.2×10^-2^ | 0.137 | |
| 40 |  |  |  | 80 | LOC285095 | 3.5×10^-2^ | | 0.120 | 120 | NPTXR | 2.4×10^-2^ | 0.138 | |
